# Effects of different rootstocks on phenolics in the skin of ‘Cabernet Sauvignon’ and widely targeted metabolome and transcriptome analysis

**DOI:** 10.1093/hr/uhac053

**Published:** 2022-03-14

**Authors:** Zhijun Zhang, Junli Sun, Shucheng Zhao, Qianjun Lu, Lizhong Pan, Baolong Zhao, Songlin Yu

**Affiliations:** Department of Horticulture, College of Agriculture, Shihezi University, Shihezi 832003, China; The Key Laboratory of Special Fruits and Vegetables Cultivation Physiology and Germplasm Resources Utilization of the Xinjiang Production and Construction Group, Shihezi University, Shihezi 832003, China; Department of Horticulture, College of Agriculture, Shihezi University, Shihezi 832003, China; The Key Laboratory of Special Fruits and Vegetables Cultivation Physiology and Germplasm Resources Utilization of the Xinjiang Production and Construction Group, Shihezi University, Shihezi 832003, China; Department of Horticulture, College of Agriculture, Shihezi University, Shihezi 832003, China; The Key Laboratory of Special Fruits and Vegetables Cultivation Physiology and Germplasm Resources Utilization of the Xinjiang Production and Construction Group, Shihezi University, Shihezi 832003, China; Department of Horticulture, College of Agriculture, Shihezi University, Shihezi 832003, China; The Key Laboratory of Special Fruits and Vegetables Cultivation Physiology and Germplasm Resources Utilization of the Xinjiang Production and Construction Group, Shihezi University, Shihezi 832003, China; Department of Horticulture, College of Agriculture, Shihezi University, Shihezi 832003, China; The Key Laboratory of Special Fruits and Vegetables Cultivation Physiology and Germplasm Resources Utilization of the Xinjiang Production and Construction Group, Shihezi University, Shihezi 832003, China; Department of Horticulture, College of Agriculture, Shihezi University, Shihezi 832003, China; The Key Laboratory of Special Fruits and Vegetables Cultivation Physiology and Germplasm Resources Utilization of the Xinjiang Production and Construction Group, Shihezi University, Shihezi 832003, China; Department of Horticulture, College of Agriculture, Shihezi University, Shihezi 832003, China; The Key Laboratory of Special Fruits and Vegetables Cultivation Physiology and Germplasm Resources Utilization of the Xinjiang Production and Construction Group, Shihezi University, Shihezi 832003, China

Dear Editor,

Cabernet Sauvignon is one of the most economically valuable and widely planted wine grape varieties. It is widely planted in Xinjiang, an important grape-producing area in China. Grafting, an ancient agricultural technique, is widely used to improve the biotic and abiotic resistance of grapes. With continuous increases in consumer requirements for grape fruit quality, the content and activity of various phenolic substances in grape skins have attracted widespread attention. An increasing number of studies have found that rootstocks can improve grape resistance while also altering quality-related substances such as sugars, acids, polyphenols, phenolic acids, stilbene, and others. In particular, antioxidant substances have attracted more and more attention [1–3].

Transcriptome sequencing (RNA-seq) has been widely used to analyze the process of grape fruit development in different varieties and the expression of genes related to grape metabolic pathways under specific environmental conditions (e.g. biotic and abiotic stresses) [4]. Widely targeted metabolomics has developed rapidly in recent years and has the advantages of high throughput, high sensitivity, wide coverage, and accurate qualitative and quantitative analysis [5]. It has been used to explore plant phenotypic differences among species and genotypes, as well as to research differences in metabolite accumulation during plant maturation and the effects of stress on plant phenotypes. Yang et al. used a broad target metabolite group to explore the synthesis of grape anthocyanins under regulated deficit irrigation (RDI) and found that the content of most monomeric anthocyanins in the RDI group increased, and the proportion of malvidin increased [6]. Similarly, Zhang et al. used metabolomics to show that different rootstocks can alter the content of metabolites such as organic acids and polyphenols in the seeds of ‘Cabernet Sauvignon’ [7]. Although the effects of rootstocks on phenolic substances in grape fruit have been reported, few studies have reported on the metabolic regulation and enrichment of phenolic substances in grape skin, especially using extensive, high-throughput, and systematic research. A combination of transcriptomics and widely targeted metabolomics can comprehensively characterize gene expression and metabolite contents in samples, more completely revealing the related metabolic pathways and molecular mechanisms of biological processes [6, 8, 9].

Figure 1A1-A6 shows the contents of total polyphenols, total anthocyanins, total flavonoids, and resveratrol and the antioxidant activities of DPPH and ABTS in the skins of six rootstock combinations of Cabernet Sauvignon grapes over two consecutive years (2018 and 2019). In terms of phenolic contents and antioxidant activity, the CS/140R rootstock combination had the highest levels of total polyphenols [28.82 ± 1.10 (mg·g^−1^ FW), 27.73 ± 0.72 (mg·g^−1^ FW)], total flavonoids [24.77 ± 0.83 (mg·g^−1^ FW), 26.71 ± 0.23 (mg·g^−1^ FW)], resveratrol [4.51 ± 0.11 (μg·g^−1^ FW), 3.96 ± 0.04 (μg·g^−1^ FW)], DPPH [1.10 ± 0.031 (mmol·g^−1^), 1.06 ± 0.014 (mmol·g^−1^)], and ABTS [1.38 ± 0.025 (mmol·g^−1^), 1.23 ± 0.025 (mmol·g^−1^)] in the two years, and these levels were significantly higher than those of the self-grafted combination (CS/CS). The total anthocyanin content [1.89 ± 0.585 (mg·g^−1^ FW), 2.25 ± 0.18 (mg·g^−1^ FW)] in the CS/SO4 combination was the highest but did not differ significantly from that of CS/140R. The total phenolic content, total anthocyanin content, and DPPH and ABTS antioxidant activities of the CS/1103P rootstock combination were slightly lower than those of the self-grafted combination (CS/CS), although this difference was not significant. The total amounts of phenolic substances (except anthocyanins) and the antioxidant activity in the grape skin of the CS/140R rootstock combination were stable and were the highest of all the treatments for two consecutive years, significantly higher than those of CS/CS.

**Figure 1:**
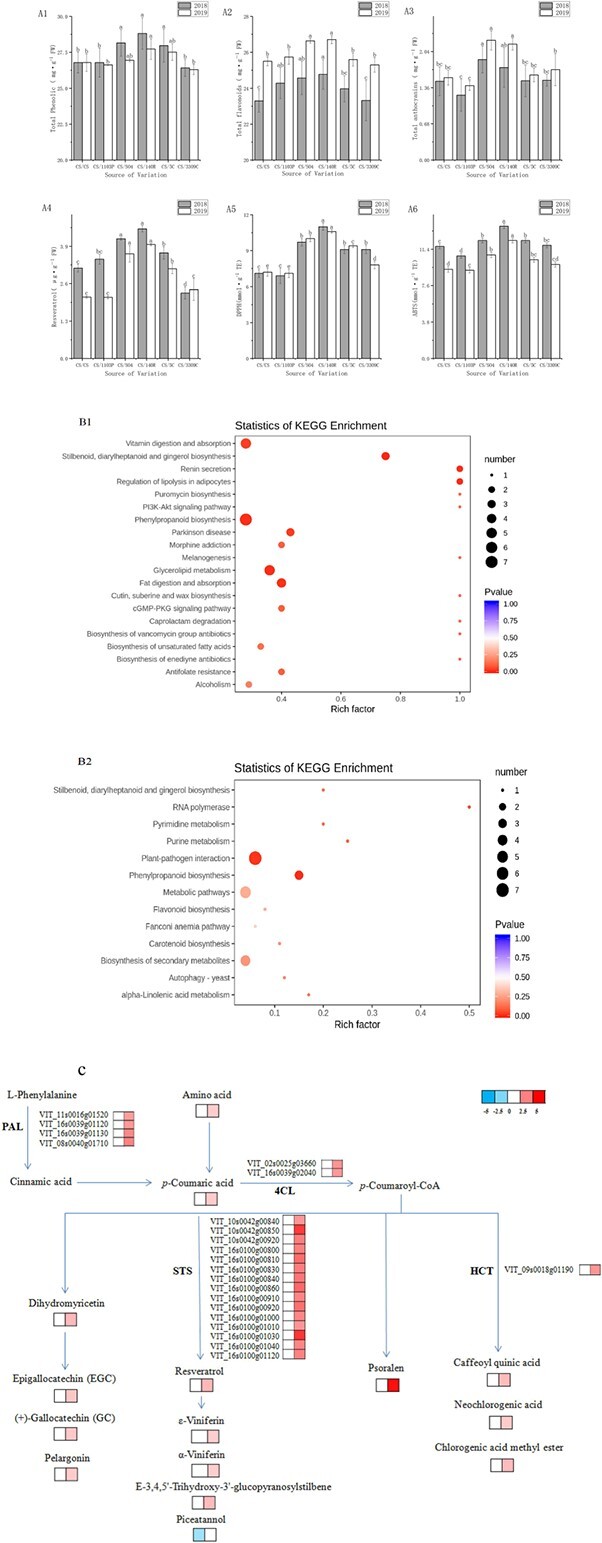
Phenolics, antioxidants, pathway analysis, and log_2_(fold-change) values of differentially expressed genes and differentially abundant metabolites in ‘Cabernet Sauvignon’ (CS) grape skins from CS/CS and CS/140R graft combinations. A1, Total phenolics; A2, total flavonoids; A3, total anthocyanins; A4, resveratrol; A5, DPPH; A6, ABTS. Different letters within a row indicate significant differences (p ≤ 0.05) among treatments for the same vintage. B1–B2, Kyoto Encyclopedia of Genes and Genomes (KEGG) pathway analysis of metabolites (B1) and transcripts (B2). C, log_2_(fold-change) values of differentially expressed genes and contents of differentially abundant metabolites. The block near each gene or metabolite is the log_2_(fold-change) of the gene or metabolite between CS/CS and CS/140R. PAL, phenylalanine ammonia-lyase; 4CL, 4-coumarate CoA ligase; STS, stilbene synthase; HCT, hydroxycinnamoyl transferase.

Widely targeted metabolomics and transcriptomics were used to investigate the pathways that regulated the synthesis of phenolic compounds in ‘Cabernet Sauvignon’ grape skins on 140R rootstocks. As shown in Fig. 1B1 and B2, biological pathway analysis was performed using the KEGG database to identify the pathways that were significantly enriched in differentially abundant metabolites (Figure 1B1) and differentially expressed genes (DEGs) (Figure 1B2). The KEGG annotation and enrichment data for benzophenone, flavonoid, and anthocyanin biosynthesis pathways in grape skins of the CS/140R rootstock combination showed that the use of 140R rootstock significantly increased the biosynthesis of phenylpropanoids, stilbenoids, and flavonoids compared with CS/CS.

As shown in Figure 1C, the grafting treatment increased the expression levels of four PAL genes (VIT_11s0016g01520, VIT_16s0039g01120, VIT_16s0039g01130, and VIT_08s0040g01710) by 2.08-, 2.04-, 2.26-, and 2.33-fold and increased the expression of two 4CL genes (VIT_02s0040_25g16s00 and VIT2) 2.01-fold. HCT increased the expression of VIT_09s0018g01190 2.08-fold, and STS increased the expression of fifteen genes (VIT_10s0042g00840, VIT_10s0042g00850, VIT_10s0042g00920, VIT_16s0100g00800, VIT_16s0100g00810, VIT_16s0100g00830, VIT_16s0100g00840, VIT_16s0100g00860, VIT_16s0100g00910, VIT_16s0100g00920, VIT_16s0100g01000, VIT_16s0100g01010, VIT_16s0100g01030, VIT_16s0100g01040, and VIT_16s0100g01120) 2.07-, 3.83-, 2.44-, 2.42-, 2.77-, 2.42-, 2.56-, 2.79-, 2.33-, 2.67-, 2.31-, 2.08-, 3.92-, 2.31-, and 2.38-fold.

The CS/140R grafting combination increased the contents of L-tyrosine 3.47-fold; 4-coumaric acid 2.13-fold; organic acids and their derivatives (caffeoylquinic acid, neochlorogenic acid, and chlorogenic acid methyl ester) 2.6-, 2.08-, and 2.53-fold; and flavonoids and anthocyanins (dihydromyricetin, epigallocatechin (EGC), (+)-gallocatechin (GC), and pelargonin) 2.60-, 2.20-, 2.16-, and 2.08-fold. With the exception of piceatannol (downregulated 3.70-fold), other stilbene substances (resveratrol, ε-viniferin, α-viniferin, and E-3,4,5′-trihydroxy-3′-glucopyranosylstilbene) were all upregulated in the CS/140R combination. The CS/140R grafting combination increased the content of the coumarin psoralen in grape skin 26.20-fold, and there was almost none of this substance in self-grafted plants (CS/CS).

In summary, we identified the rootstock combination CS/140R as having the highest levels of skin antioxidants among six rootstock combinations of Cabernet Sauvignon grapes. Widely targeted metabolomics combined with transcriptomic analysis was used to investigate the mechanisms by which grape rootstocks influenced the synthesis of phenolic compounds in the skin of Cabernet Sauvignon grapes. The results showed that rootstocks can enhance the contents of phenolic acids, flavonols, anthocyanins, stilbene metabolites, and related synthases in grape skin. Changes in gene expression increased the antioxidant capacity of the skin. These results provide insights into the composition and value of this variety’s skin, providing a theoretical basis for the development and utilization of functional grape metabolites.

## Supplementary Material

Web_Material_uhac053

## Data Availability

Data generated or analyzed during this study are included in this article and its supplementary information files.
