# Supplementary material for: Effects of different rootstocks on phenolics in the skin of ‘Cabernet Sauvignon’ and widely targeted metabolome and transcriptome analysis
Source: Hortic Res. 2022 Mar 14;9:uhac053. doi: 10.1093/hr/uhac053 (PMC9154070; doi:10.1093/hr/uhac053)
Supplement: Web_Material_uhac053 [file web_material_uhac053.zip › SUPPLEMENTAL INFORMATION.docx]

***Effects of Different Rootstocks on Phenolics in Skin of ‘Cabernet Sauvignon’ and Widely Targeted Metabolome and Transcriptome Analysis***

Zhijun Zhang^1,2*^, Junli Sun^1,2*^, Shucheng Zhao^1,2^, Qianjun Lu^1,2^, Lizhong Pan^1,2^, Baolong Zhao^1,2^ and Songlin Yu^1,2^

^1^ Department of Horticulture, College of Agriculture, Shihezi University, Shihezi 832003, P.R. China

^2^ The Key Laboratory of Special of Fruits and Vegetables Cultivation Physiology and Germplasm Resources Utilization of the Xinjiang Production and Construction Group, Shihezi University, Shihezi 832003, P.R. China

*These authors contributed equally to this paper.

Correspondence:

Baolong Zhao

E-mail: zblgrape@163.com

Phone No: +86 18009935729

Songlin Yu

E-mail: songlin8900@sina.com

Phone No: +86 13999327397

**Supplementary Information**

**Materials and Methods**

**Supplementary Figures**

**Supplementary Table**

**Supplementary references**

Materials and Methods

**Materials and methods**

**Plant materials and experimental treatments**

Cabernet Sauvignon (CS) was used as the scion. Rootstock varieties with excellent resistance were selected: rootstocks SO4 and 5C (*Vitis berlandieri × Vitis riparia*), rootstocks 140R and 1103P (*V. berlandieri × V. rupestris*), and rootstock 3309C (*V. riparia × V. rupestris*). The combination of rootstock were CS/1103P, CS/SO4, CS/140R, CS/5C, and CS/3309C. CS/CS (self-rooted grafting seedlings) was used as the control.

These were grafted and planted (0.5 m apart) in blocks in the field at the Experimental Station of the Agricultural College of Shihezi University in Xinjiang, China (86°06’ N, 44°32’ E). The same cultivation and management methods were used for planting to ensure the yield of each group was similar. Approximately 200 fully mature berries were randomly picked from 10 vines (repeated in triplicate for each treatment). The berries were rinsed with deionized water, quickly skined, frozen in liquid nitrogen, and stored at −80°C.

**Determination of maturity indicators**

The soluble sugar content was determined using the anthrone colorimetric method. The method of Tian et al. ^1^ was used to improve the content. Fresh berry pulp was then mixed and filtered. Next, 0.5 ml of the filtrate was added to 250 ml of 80% ethanol solution and boiled for 30 min. After cooling to a constant volume to 250 ml, 1 ml of diluent was mixed with 0.2% 4 ml of anthrone and sulfuric acid solution for 20 minutes. The resulting mixture was measured using a UV-visible spectrophotometer (UV-2600; Shimadzu, Japan). The result is given in mg glucose/g FW. Next, the filtrate was titrated with 0.1 M NaOH to an end point of pH 8.3. The results are expressed as mg tartaric acid per gram of FW.

**Determination of phenolics and antioxidants**

The total phenolic content was determined using the Folin-Ciocalteu method described by Myrtsi. ^2^ The grape skin were ground and extracted with a methanol/water (50:50, v/v) solution, sample (adequately diluted), Folinreagent (0.62 M), sodium carbonate solution 4% (w/w), and ultrapure water (1:4:4:4). After extraction in the dark for one hour, absorbance was measured at λ = 765 nm using a UV-visible spectrophotometer, and the results were expressed in milligram gallic acid equivalents (mg GAE)/mL.

The flavonoids were determined by the aluminum nitrate method for specific experimental procedures, as described by Saeed et al.^3^ The grape skin were collected and ground, to which an appropriate volume of extract (30% methanol, 5% NaNO_2_, and 10% AlCl3·6H_2_O) was added. After leaching for 5 min, 1 ml of 1 M NaOH was added. The UV-visible spectrophotometer was used to measure the flavonoid content at 500 nm, and the result was expressed in milligrams of rutin equivalent per gram of fresh fruit, recorded as mg RE·g FW^-1^.

The anthocyanin content was measured using the pH differential method. ^4^ The grape skin were added to 10 ml of 1% hydrochloric acid methanol for 4 h in an incubator at 32°C in the dark. The absorbance was measured at 553 nm and 600 nm using the UV-visible spectrophotometer.

The resveratrol content was determined by HPLC. The grape skin were ground and mixed with methanol at 1:10, followed by ultrasonic extraction for 30 min and centrifugation at 12000 rpm for 10 min. The supernatant was filtered through a 0.45-µm filter membrane in a chromatographic bottle. HPLC (LC-2010AHT; Shimadzu, Japan) with an injection volume of 10 µL was measured at 553 nm and 600 nm. The mobile phases were acetonitrile and water (containing 0.2% phosphoric acid), and the gradient elution and specific elution methods were performed as described by Zhou et al.^5^

**Determination of antioxidant capacity**

The DPPH free radical scavenging ability was measured as reported by Xu et al.^6^ The extract or reference substance Trolox solution and DPPH working solution were shaken at 1:10, and reacted in the dark at room temperature for 1 h. Measurements were obtained using the UV-visible spectrophotometer at a wavelength of 517 nm. The iron reduction antioxidant capacity (FRAP) was measured as the extract or reference substance Trolox solution and DPPH working solution were shaken at 1:10. The reaction was performed in the dark at room temperature for 10 min and measured with a UV-visible spectrophotometer at a wavelength of 734 nm. For ABTS and FRAP analysis, the free radical scavenging activity of the sample was expressed as Trolox equivalent antioxidant capacity, and the calculation result was expressed in millimoles of Trolox equivalent per gram of fresh fruit (mmol·g^-1^ TE).

**Widely targeted metabolome**

According to Gaihua et al. and Wang et al., ^7, 8^ the freeze-dried grape skin samples were crushed for 1.5 min at 30 Hz using a mixer with zirconia beads (MM 400; Retsch, Germany). Next, 100 mg of the powder was weighed and extracted with 1.0 ml methanol water (30:70 v/v, 4°C) for 12 h in the dark. After centrifugation at 10000 × *g* for 10 min, the extract was adsorbed by CNWBOND Carbon-GCB SPE Cartridge (ANPEL, Shanghai, China), filtered with a SCAA-104 membrane (0.22 µm) (ANPEL, Shanghai, China), and analyzed by LC-MS.

**LC-ESI-MS/MS system**

For HPLC (Shim-pack UFLC SHIMADZU CBM30A system) and MS (Applied Biosystems analysis sample extract 6500 Q trap), a C18 column (1.8 µm, 2.1 mm × 100 mm, Waters ACQUITY UPLC HSS T3) was used. Water, acetonitrile, and 0.04% acetic acid (v/v) were used as the mobile phases A and B, respectively. The column temperature was 40°C and the flow rate was 0.4 mL/min. An injection volume of 2 µL was used. Gradient elution: 0-11 minutes, 95 A: 5 B (v:v); 11-12 minutes, 5 A: 95 B (v:v); and 12.1-15 minutes, 95 A: 5 B (v:v). The system was run in positive ion mode and controlled by Analyst 1.6 software (AB Sciex). The ESI conditions included turbo spray, 5500 V, and 550°C. The pressures of ion source gas I (GSI), ion source gas II (GSII), and curtain gas (CUR) were 55, 60, and 25 psi, respectively, and the collision gas (CAD) pressure was set higher.

In the QQQ and LIT modes, 10 and 100 μmol/L polypropylene glycol solutions were used for instrument tuning and mass calibration, respectively. The QQQ scan was obtained as an MRM experiment, and the collision gas (nitrogen) was set to 5 psi. The integration and calibration of chromatographic peaks was performed using MultiQuant version 3.0.2 (AB SCIEX, Concord, Canada). The corresponding relative metabolite content was expressed as the integral of the chromatographic peak area. ^9.10^ Multivariate statistical analysis (software package SIMCA-P (version 13.0) and R “ropls” package) was used to perform principal component analysis (PCA), orthogonal partial least square discriminant analysis (OPLS-DA), and other chemometric principles and multivariate statistical analysis of the data.

**Transcriptome analysis**

Trizol reagent (Invitrogen, Life Technologies) was used to isolate total RNA from grape skin, as described by Zhang et al. ^4^ The UltraTM RNA Library Prep Kit (Illumina, San Diego, CA, USA) was used for library construction. The resulting library was sequenced on the Illumina HiSeq 4000 platform, and 150-bp paired-end reads were generated. Fastp (version 0.19.3) was used to filter the original data, and HISAT (version 2.1.0) was used to build an index. The clean reads were compared to the reference genome. The reference genome data were obtained from the Ensembl database (http://www.ensembl.org/).

FeatureCounts (version 1.6.2) was used to calculate the gene alignment, and then calculated the FPKM of each gene based on the gene length. DESeq2 v1.22.1 was used for differential expression analysis between the two groups.^11^

**Differential gene screening**

DESeq was used to analyze the differential expression of the genes. The conditions for differentially expressed genes were as follows: expression difference multiple |log2FoldChange| > 1 and *p* < 0.05. On this basis, GO and KEGG were further used to enrich and analyze the differences in expression.^12^

Vector NTI 10 software was used to design qRT-PCR primers according to the coding sequences of the nine candidate genes selected. The designed primers were synthesized by China Shanghai Bioengineering Co., Ltd. (Shanghai, China) and the primer sequence (Table S2).

**Supplementary Figures 1, 2**

**
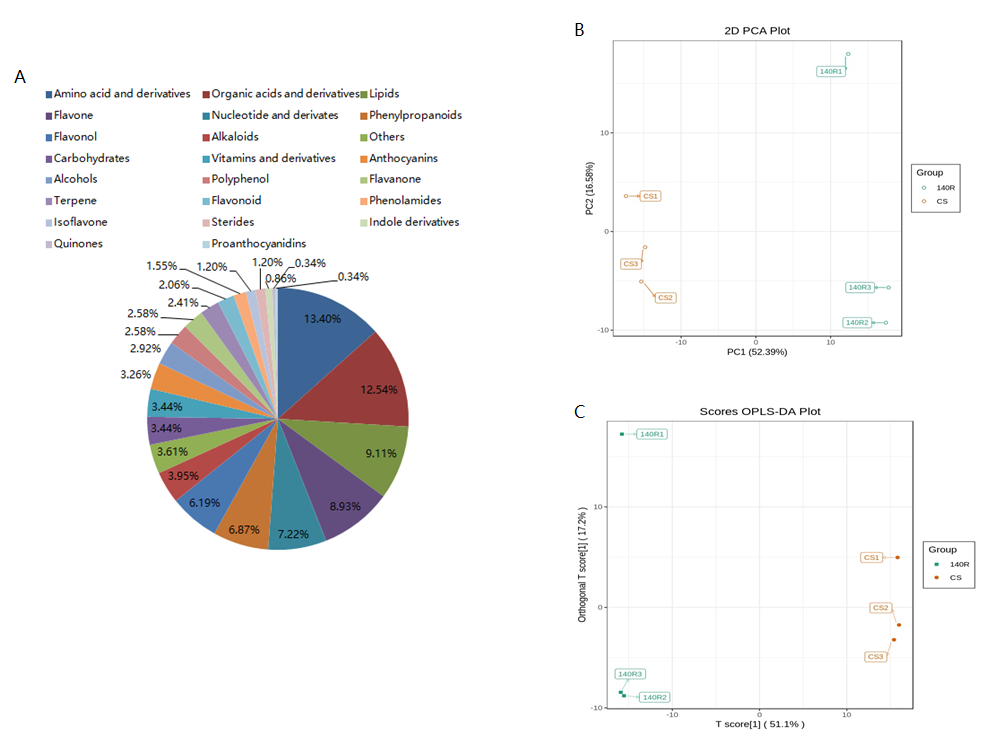
Supplementary Figures 1 Qualitative and quantitative analysis of the metabolomics data of ‘Cabernet Sauvignon’ (CS) skin from CS/CS and CS/140R.** (A) Component analysis of the identified metabolites. (B) Principal components analysis (PCA) scores of metabolites. (C) Orthogonal-partial least squares discrimination analysis (OPLS-DA) score plot of metabolites.


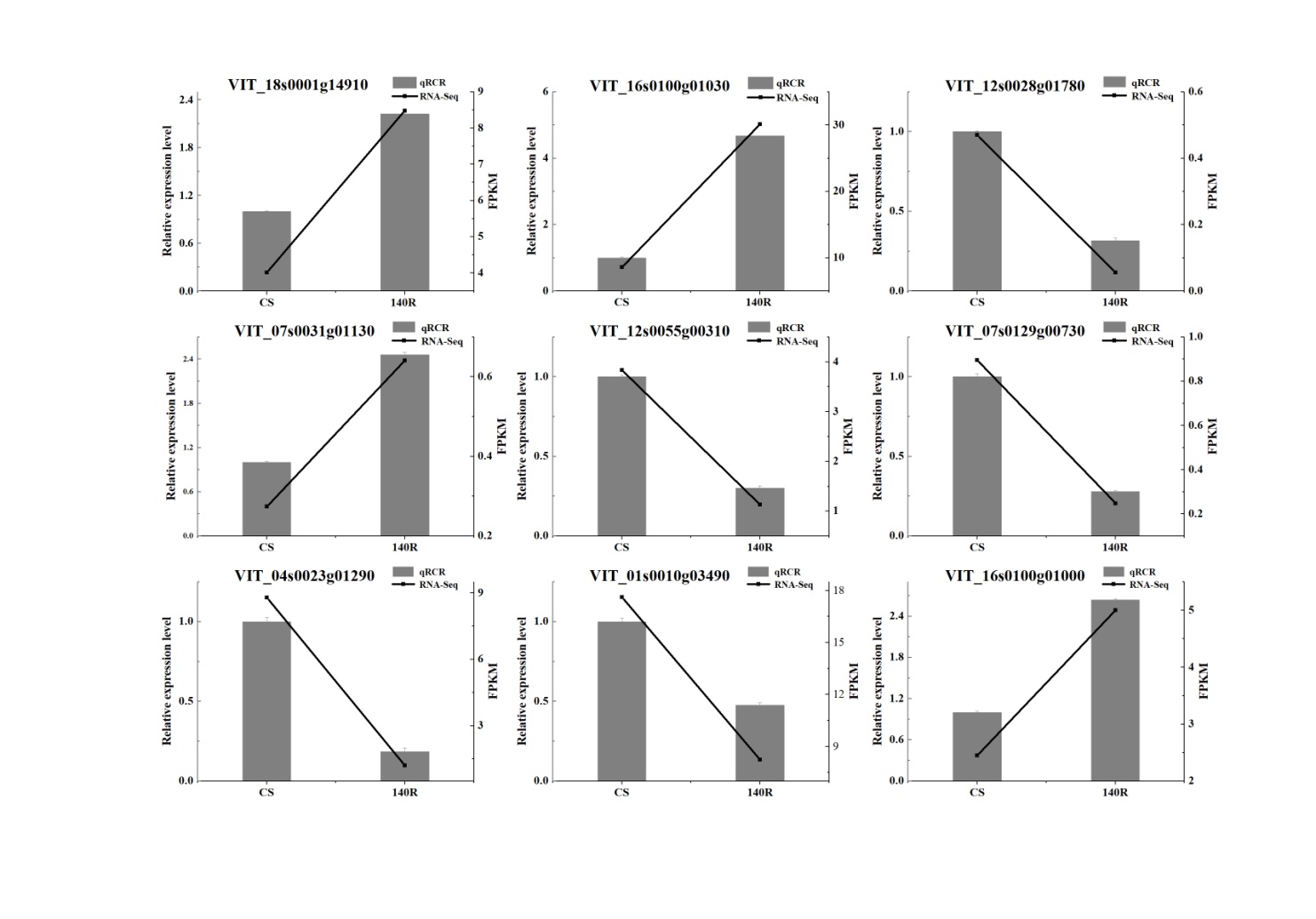


**Supplementary Figures 2 Comparison of quantitative real-time-PCR (qRT-PCR) and transcriptome sequencing (RNA-Seq) results for nine candidate genes.**

**Supplementary Table 1, 2**

**Supplementary Table 1 Maturity index of ‘Cabernet Sauvignon’ skin from different scion-rootstock combinations**

**Supplementary Table 2 Primers used in the qRT-PCR**

| Gene ID | Primes | Sequences(5’→3’) |
| --- | --- | --- |
| VIT_04s0044g00580 | Actin1 F | CTTGCATCCCTCAGCACCTT |
|  | Actin1 R | TCCTGTGGACAATGGATGGA |
| VIT_18s0001g14910 | Probable cinnamyl alcohol dehydrogenase 6 F | TGCTGAAGGTGAACGGGACAATG |
|  | Probable cinnamyl alcohol dehydrogenase 6 R | CACATCCATCATCTCCTGCGTCTC |
| VIT_16s0100g01030 | Stilbene synthase 3 F | TTGAACGACCACTCTTCCAGCTTG |
|  | Stilbene synthase 3 R | GTCCCACCTCACGTAAGTTTCCTG |
| VIT_12s0028g01780 | Berberine bridge enzyme-like 13 F | AGAGGTGGTGGAGGAGCAAGC |
|  | Berberine bridge enzyme-like 13 R | ACGGTCACAGTGGATGGGACAG |
| VIT_07s0031g01130 | L-ascorbate oxidase F | TCAAGCAGCAGAGGCAAGAATCAG |
|  | L-ascorbate oxidase R | TCAAGCAGCAGAGGCAAGAATCAG |
| VIT_12s0055g00310 | Anthocyanidin 3-O-glucosyltransferase 2 F | CACTCGCTCCAACTCGGTTCG |
|  | Anthocyanidin 3-O-glucosyltransferase 2 R | TGCAACAGGAAGCCAAGAGAAGC |
| VIT_07s0129g00730 | Cytochrome P450 F | TCTGGTCAGTCCCGTTCAATTTGC |
|  | Cytochrome P450 R | CTCAAGTTCCTGCCTCTTCTCGTG |
| VIT_04s0023g01290 | Anthocyanidin 3-O-glucosyltransferase 5 F | TGGAGGACTCGGAAGGGAATGC |
|  | Anthocyanidin 3-O-glucosyltransferase 5 R | GATCGGCTCATCATCTGGCAATCC |
| VIT_01s0010g03490 | Flavonoid 3',5'-methyltransferase-like F | TCATTGCCGATGGGAACGAAGAAG |
|  | Flavonoid 3',5'-methyltransferase-like R | TTCAGCAGCAGCTCATGGTAGTTG |
| VIT_16s0100g01000 | Stilbene synthase 4 F | CGACAGGTGAAGGATTGGATTGGG |
|  | Stilbene synthase 4 R | AACGCTATGGATCACAACGGTCTC |

**Supplementary references**

1. Tian, S.G., Zhou, X.Y., Gong, H.Y., Ma, X.M. & Zhang, F. Orthogonal test design for optimization of the extraction of polysaccharide from Paeonia sinjiangensis KY Pan. *Pharmacogn Mag* **7**, 4-8 <http://dx.doi.org/10.4103/0973-1296.75874> (2011).

2. Myrtsi, E.D., Koulocheri, S.D., Iliopoulos, V. & Haroutounian, S.A. High-Throughput Quantification of 32 Bioactive Antioxidant Phenolic Compounds in Grapes, Wines and Vinification Byproducts by LC-MS/MS. *Antioxidants* **10**, 20 1174 <http://dx.doi.org/10.3390/antiox10081174> (2021).

3. Saeed, N., Khan, M.R. & Shabbir, M. Antioxidant activity, total phenolic and total flavonoid contents of whole plant extracts Torilis leptophylla L. *Bmc Complem Altern M* **12**, <http://dx.doi.org/Artn> 22110.1186/1472-6882-12-221 (2012).

4. Zhang, Z.J. *et al.* Effects of 5-aminolevulinic acid on Anthocyanin synthesis in *Vitis Vinifera 'Crimson Seedless'* grapes at the transcriptomics level. *J. Horticult. Sci. Biotechnol.* 11 <http://dx.doi.org/10.1080/14620316.2021.1930589>.

5. Zhou, Q. *et al.* Resveratrol derivatives in four tissues of six wild Chinese grapevine species. *New Zeal J Crop Hort* **43**, 204-213 <http://dx.doi.org/10.1080/01140671.2015.1010547> (2015).

6. Xu, L.L., Yue, Q.Y., Bian, F.E., Zhai, H. & Yao, Y.X. Melatonin Treatment Enhances the Polyphenol Content and Antioxidant Capacity of Red Wine. *Hortic Plant J* **4**, 144-150 <http://dx.doi.org/10.1016/j.hpj.2018.05.004> (2018).

7. Qin, G.H. *et al.* Diversity of metabolite accumulation patterns in inner and outer seed coats of pomegranate: exploring their relationship with genetic mechanisms of seed coat development. *Hortic. Res.-England* **7**, 1410 <http://dx.doi.org/10.1038/s41438-019-0233-4> (2020).

8. Wang, Z., Cui, Y., Vainstein, A., Chen, S. & Ma, H. Regulation of Fig (*Ficus carica L.*) Fruit Color: Metabolomic and Transcriptomic Analyses of the Flavonoid Biosynthetic Pathway. *Front Plant Sci* 8, 1990 <http://dx.doi.org/10.3389/fpls.2017.01990> (2017).

9. Carlos, F. et al. Signature-discovery approach for sample matching of a nerve-agentprecursor using liquid chromatography-mass spectrometry, XCMS, and chemometrics. *Analytical Chemistry*, **82**, 4165-4173 <http://dx.doi.org/10.1021/ac1003568> (2010)

10. Chen, W. et al. A Novel Integrated Method for Large-Scale Detection, Identification, and Quantification of Widely Targeted Metabolites: Application in the Study of Rice Metabolomics. *Mol. Plant.* **6**, 1769-1780 <http://dx.doi.org/10.1093/mp/sst080> (2013).

11. Love M., Huber W., & Anders S. This Provisional PDF corresponds to the article as it appeared upon acceptance. Fully formatted Moderated estimation of fold change and dispersion for RNA-seq data with DESeq2. *Genome Biol*. **15**, 550 [http://dx.doi.org/10.1186/PREACCEPT-8897612761307401 (2014)](http://dx.doi.org/10.1186/PREACCEPT-8897612761307401%20(2014))

12. Yu G. et al. clusterProfiler: an R package for comparing biological themes among gene clusters. *Omics- J Integr Biol*. **16**,284-287 http://dx.doi.org/10.1089/omi.2011.0118 (2012)
